# Therapeutic Exercise Interventions through Telerehabilitation in Patients with Post COVID-19 Symptoms: A Systematic Review

**DOI:** 10.3390/jcm11247521

**Published:** 2022-12-19

**Authors:** Carlos Bernal-Utrera, Gines Montero-Almagro, Ernesto Anarte-Lazo, Juan Jose Gonzalez-Gerez, Cleofas Rodriguez-Blanco, Manuel Saavedra-Hernandez

**Affiliations:** 1 Physiotherapy Department, Faculty of Nursing, Physiotherapy and Podiatry, University of Seville, 41004 Sevilla, Spain; 2 Fisiosur I+D Research Institute, 04630 Garrucha, Spain; 3 Doctoral Program in Health Sciences, University of Seville, 41004 Seville, Spain; 4 Department Nursing, Physiotherapy and Medicine, University of Almeria, 04120 Almeria, Spain

**Keywords:** exercise, telerehabilitation, COVID-19, physiotherapy, quality of life

## Abstract

The worldwide incidence of COVID-19 has generated a pandemic of sequelae. These sequelae require multidisciplinary rehabilitative work to address the multisystemic symptoms that patients will present with now and in the future. The aim of the present systematic review is to analyze the current situation of telerehabilitation in patients with COVID-19 sequelae and its effectiveness. Searches were conducted on the following databases: PubMed, Scopus, PEDro, and Web of Science (WOS). There was no complete homogeneity among the five selected articles, so we differentiated two clinical subgroups for the clustering of outcome measures: (group one) patients with post-discharge symptoms and (group two) patients with permanent symptoms or “long COVID-19” defined as persistent symptoms > 2 months. For group one, post-discharge sequelae, improvements were obtained in cardiovascular parameters, and physical test studies in group two presented very favorable results in all the cardiorespiratory measures and physical tests evaluated. Telerehabilitation through therapeutic exercise based on mixed protocols of aerobic, respiratory, and low-load strength exercises appear to be an effective and safe strategy for the recovery of short- and long-term post-COVID-19 sequelae.

## 1. Introduction

The worldwide incidence of COVID-19 has generated a pandemic of sequelae. These sequelae require multidisciplinary rehabilitative work to address the multisystemic symptoms that patients will present with now and in the future [1,2]. In addition to primary pulmonary pathology, which is divided into different stages and can lead to pulmonary fibrosis [3,4], pathologies in the cardiovascular [5,6], gastrointestinal [7], neurological [8], musculoskeletal [9], dermatological [10], and ophthalmic regions [11] as well as postviral fatigue and cognitive impairment [12] are described as sequelae in the current literature.

During the coronavirus pandemic, the need for maintenance of organ functions through rehabilitation and its continuation has put telerehabilitation programs in a status unusually seen before [13], not only during the acute phase but also in the postviral phase [14,15,16,17,18]. Such telerehabilitation demands and the consequent implementation of physiotherapy staff and training continue to this day [19,20]. Studies prior to the COVID-19 pandemic analyzed not only the therapeutic benefits of cardiac, neurological, or musculoskeletal telerehabilitation but also the cost savings for both healthcare providers and patients compared to traditional inpatient or face-to-face rehabilitation [21]. Telerehabilitation is positioned as a real alternative to in-person rehabilitation in the context of cardiac and pulmonary rehabilitation among others [22]. Not only physical but also cognitive virtual reality exercises using telerehabilitation have been shown to be effective and safe for the patient’s post COVID-19 condition [23]. The satisfaction perceived by patients in different studies during the confinement phase and afterwards indicates that telerehabilitation can and should be an element to be considered for the comprehensive rehabilitation of patients in the acute phase and with sequelae of COVID-19 [24,25].

The aim of the present systematic review is to analyze the current situation of telerehabilitation in patients with COVID-19 sequelae and its effectiveness.

## 2. Materials and Methods

This systematic review was registered in PROSPERO with the number CRD42022360887 and was made following the recommendations of PRISMA [26].

### 2.1. Identification and Selection of Studies

Searches were conducted on the following databases: PubMed, Scopus, PEDro, and Web of Science (WOS). Searches lasted until 30 September 2022 by two independent reviewers (GMA) and (CBU) according to the PICO question. MESH terms were used, adapting the search strategy to the different databases requirements, search strategy for each database can be seen in Table 1. Search filters were used when available on the different databases. The following filters were used: time (2019–2022), article, human, and scientific journal.

The inclusion and exclusion criteria were defined using the PICOS question acronym, (P: Population, I: Intervention, C: Comparison, O: Outcomes, S: Study Design); a description of PICOS question can be seen in Table 2.

Inclusion criteria:

- Post COVID-19 patients with symptoms.

Exclusion criteria:

- Exclusive groups of Intensive Care Unit patients.
- Any permanent dysfunction generated by complications of treatments or other diseases and not directly generated by COVID-19.

### 2.2. Data Extraction

Two reviewers (GMA and CBU) searched titles and abstracts using the inclusion and exclusion criteria. For those studies that met the requirements, full text was obtained. If there were any doubts about a study meeting the requirements assessing the title and abstract, full text was consulted. If needed, the original author of the text would be contacted. Full text was applied the same eligibility criteria.

The articles were included in this systematic review if both reviewers agreed. In case of disagreement, they would meet and discuss to reach an agreement. If after that, they still failed to reach an agreement, a third independent reviewer (CRB) was consulted to determine the inclusion or exclusion of the text applying eligibility criteria. The reviewers of this systematic review were not blinded to the titles of the journals nor the author’s names of the texts.

### 2.3. Risk of Bias

The risk of bias and quality of studies were assessed using the PEDro Scale for RCTs which is an 11-item scale to assess internal validity (item 2–9), whether studies have sufficient statistical information (item 10), and to interpret results (item 11). PEDro scores of 0–3 are considered “poor’, 4–5 ‘fair’, 6–8 ‘good’, and 9–10 ‘excellent’. The first item aims to assess external validity, but it does not account for the total score [27]. The Jadad Scale is a 5-point scale to assess quality of RCTs; it consists of 3 items: the first item evaluates randomization, the second one evaluates blinding, and the third one evaluates the losses on follow up [28]. Non-RCT study designs were assessed using the Newcastle–Ottawa Scale (NOS) where three factors were considered to score the quality of the included studies: (1) selection, including representativeness of the exposed cohort, selection of the non-exposed cohort, ascertainment of exposure, and demonstration that at the start of the study, the outcome of interest was not present; (2) comparability, assessed on the basis of study design and analysis, and whether any confounding variables were adjusted for; and (3) outcome, based on the follow-up period and cohort retention, and ascertained by independent blind assessment, record linkage, or self-report. NOS contains 8 items within 3 domain and the total maximum score is 9. A study with score from 7–9, has high quality; 4–6, fair quality; and 0–3, poor quality [29].

Two independent reviewers (EAL and MSH) individually applied the different scales to the selected studies. In case of difference between scores, both reviewers would discuss this discrepancy in order to reach an agreement. If they could not reach a consensus, a third independent reviewer (CBU) would participate in the discussion to reach an agreement about the score. The intraclass correlation coefficient for the PEDro, Jadad, and NOS were evaluated.

### 2.4. Data Synthesis

A narrative synthesis of all the data extracted was performed. Our aim was to synthesize and evaluate the applied telerehabilitation methods and verify their effectiveness in permanent symptoms of physical and psychological condition. Only quantitative results were considered, and a formal synthesis was generated in a narrative manner between all the results extracted.

At least five studies that met the inclusion/exclusion criteria were required for its completion, we set a minimum of five articles to obtain a sufficient amount of information to ensure a quality synthesis, in addition, they were required to meet minimum quality criteria based on the PEDro and Ottawa scales, fulfilling at least 50% of the evaluation items. The assessment of bias should generate an intraclass correlation coefficient of at least 60% between the two assessors.

We considered reasons for heterogeneity that are related to participants, intervention, outcomes, or trial settings. A subdivision of results was made when we believed that some of these variables differed in a critical way.

The assessment of the certainty of the evidence should aim to consider the precision of the synthesis results (confidence interval if available), the number of studies and participants, the consistency of effects between studies, the risk of study bias, how directly the included studies address the planned question (directivity) and the risk of publication bias based on the PEDro, JADAD, and Ottawa scales, as mentioned above.

The findings of the studies presented in the tables included the key characteristics, the study design, the sample size, and the risk of bias, we considered these key sections, since they could affect the interpretation of the data.

## 3. Results

**Figure 1:**
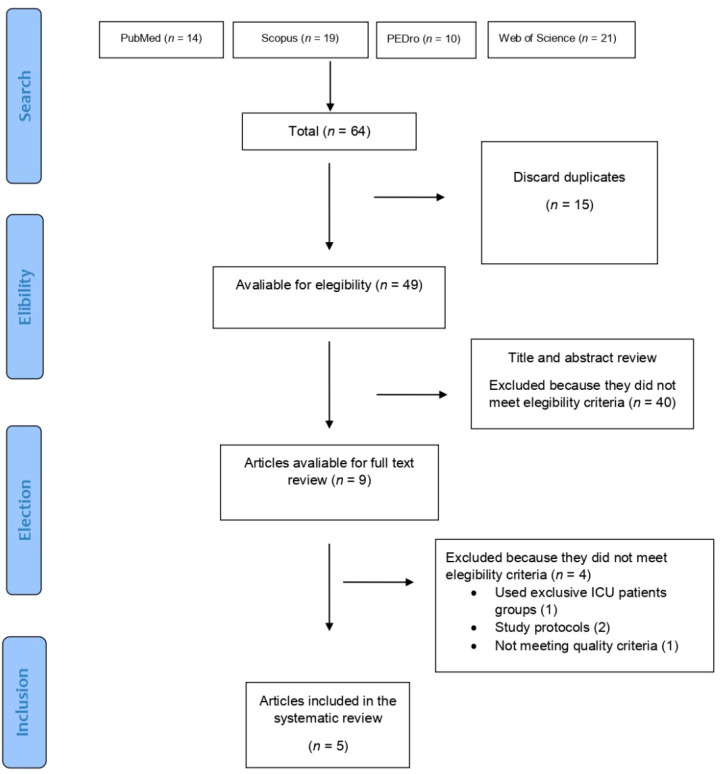
Flow chart diagram.

In total, 64 results were retrieved after the searches using combinations of the search terms. After curating the search reading title and abstract, a total of 55 articles were discarded; the main reasons for this were the article being a systematic review (fourteen), the topic not being related (twenty-six), and repeated topics (fifteen). The remaining nine articles were reviewed reading the full text thereafter, with three of them not meeting the inclusion criteria and one study not meeting the minimum quality requirements [30], leaving us with a total amount of five articles for this systematic review; study designs were variable, two of them presented a randomized controlled trial design [31,32], one presented a nonrandomized controlled trial [33], and two presented a quasi-experimental design [34,35]. The complete flow diagram can be seen in Figure 1.

### 3.1. Characteristics of Included Studies

There was no complete homogeneity among the five selected articles [31,32,33,34,35], so we differentiated two clinical subgroups for the clustering of outcome measures: group one patients with post-discharge symptoms (two studies) [31,32], and group two patients with permanent symptoms or “long COVID-19” defined as persistent symptoms > 2 months (three studies) [33,34,35]. Controlled studies use traditional rehabilitation [34], short health tips [32], and home exercise sheets [31] as a comparison. The age of the participants was not centralized in an age group, the presence of adults being a common criterion among all the articles, and the elderly population was excluded in some cases. Among the selected articles, the average age of the subjects included was around 50 years old. A summary table of all the outcome variables can be seen in Appendix A.

### 3.2. Evaluation Variables

Among the studies included, the evaluated aspects were divided into three groups; physical tests (6 Minute Walking Test and Distance (6MWT-6MWD), 60 s sit to stand test (60secSTS) Physical Performance Battery (SPPB) Time Up and Go Test (TUG); Short Form Health Survey-12-Physical Condition Score (SF-12 pCS), cardiorespiratory values (Chalder Fatigue Score (CFS-11); forced expiratory volume in 1 s (FEV-1); heart rate (HR); Modified Borg Dyspnea Scale (MBDS); Modified Medical Research Council Dyspnea Scale (mMRC); maximum voluntary ventilation (MVV); resistance exercise (RE); peak expiratory flow (PEF); end-tidal carbon dioxide pressure at rest (PetCO2 rest); at peak (PetCO2 peak); at recovery (PetCO2 recovery); St George’s Respiratory Questionnaire (SGRQ); oxygen saturation (SpO2); Fatigue Visual Analogic Scale (VAS); ventilation per unit of carbon dioxide production slope (VE/VCO2); maximal oxygen uptake (VO2 max); power at the first ventilatory threshold (VT1)) and cognitive variables (Beck Depression Inventory (BDI); Short Form Health Survey-12-Mental Condition Score (SF-12 mCS)). All studies evaluated medium-term changes (2–4 months) [31,32,33,34,35] and only one study evaluates long-term results (6 months) [32].

### 3.3. Telerehabilitation Methods

The telerehabilitation methods used were very similar, with four studies using the same method, and guided sessions supervised by a health professional via video conferencing [31,33,34,35]. Only one study opted for an unsupervised program with one weekly teleconsultation where any necessary questions were resolved [32].

### 3.4. Exercise Protocols

At least three sessions per week and at most five [35] with of a 40–60 min duration were contemplated for the therapeutic exercise programs, the duration of the programs varying between 4 and 7 weeks.

The structure of the sessions varied between the different studies; however, most studies opted for a mixed program of intervention through aerobic exercise and breathing exercises as a basis [31,32,34,35]. Two of them supplemented the intervention with strength exercises for lower limbs [32] and all muscle groups [35]. Two others supplemented the intervention with health education [31,34]. Only one study did not include breathing exercises and health education, focusing exclusively on an aerobic load-focused training program supplemented with strength [33].

### 3.5. Adverse Events

None of the studies reported adverse events of interest [31,32,33,34,35].

### 3.6. Physical and Cardiovascular Results

For group one, post-discharge sequelae, improvements were obtained in cardiovascular parameters and physical tests, with slight improvements found in one study [31] and more significant improvements found in another [32]. With respect to the control groups, telerehabilitation was only superior to the active control group in SGRQ [31], although it obtained significant changes in all physical tests when against a passive control [32]. Pulmonary function did not show significant changes compared to no intervention; both groups improved over time [32].

The studies in group two presented very favorable results in all the cardiorespiratory measures and physical tests evaluated [33,34,35], the effect sizes of the treatments vary greatly depending on whether the study was small, medium [35], or large [34]. Only one study compared its results with respect to an active control, without obtaining statistically significant improvements with respect to traditional rehabilitation, and this study was the only one that evaluated strength measures without showing changes in either group [33].

### 3.7. Cognitive Results and Quality of Life

Only two studies of those included in this review assessed psychological variables; for this question, the Beck Depression Index and SF-12 (Mental Component Score) were used, and no significant differences were observed with respect to the control groups after the intervention [31,32].

### 3.8. Quality Assessment

The average score for the PEDro Scale was 8; for the Jadad Scale, it was 3 out of 5; and for the NOS, it was 6; the global level of evidence was good for RCT and fair for NRCT. The global intraclass correlation coefficient was 0.92, individually for the PEDro, Jadad, and NOS were 0.80, 1, and 0.50, respectively. The individual study values can be seen in Table 3.

## 4. Discussion

The main objective of this review was to check the current status of telerehabilitation for post-COVID-19 sequelae. Based on the results obtained, we can state that telerehabilitation is positioned as a viable and effective option for implementation in clinical practice and as an approach to post-COVID-19 sequelae, although we consider it necessary to discuss some key points that we have identified during this review.

Firstly, we would like to emphasize that we have identified two well-differentiated groups of post-COVID-19 sequelae, the first being the short-term post-discharge sequelae, and the second being the permanent sequelae known as “long COVID”; both are sequelae of the disease and therefore were included in this review, although analyzed independently, since we observed differences in the evolution of these after the implementation of different telerehabilitation protocols. From the point of view of intervention, we did not generate any subdivision, since all the authors implemented a very similar therapeutic exercise protocol, based mainly on aerobic and respiratory exercise, and in some cases, complemented by low-load strength exercises and health education; however, we do not believe that the differences are sufficient enough to be remarkable, but we consider all of them to be valid and correct protocols of clinical recovery.

All the studies analyzed show improvements in the cardiorespiratory parameters and physical tests evaluated, such as dyspnea, heart rest, 6MWT, and 30STS, and the effectiveness of the various therapeutic exercise protocols implemented through telerehabilitation seems clear. Only one study evaluated patients at the spirometry level [32], and did not obtain favorable results, although these patients had immediate sequelae and therefore perhaps did not present sufficient respiratory deficits to objectify changes. It has been observed that cardiopulmonary impairments improve over time; moreover, these cardiopulmonary measures do not always associate with lung function, because some of the subjective measures related to activity could gain relevance [36]. From a cognitive point of view, the two studies that evaluated some variables agree that there is no change with respect to the control at post-disease follow-up [31,32]. This does not exclude the possibility of cognitive deterioration seen in post-COVID-19 patients [37,38]. However, rehabilitation and telerehabilitation programs could help in preventing or lessening the impact of those sequelae. Physical exercise has been positioned as a pill to boost cognitive and executive functions in mild cognitive impairment [39,40]; nonetheless, the underlying mechanisms of cognitive impairment in post-COVID-19 patients are unclear [41], although it seems that neuroinflammation is one of the determining factors. Given the anti-inflammatory effect of exercise on neuroinflammation [42], it would be interesting to study the effect of exercise in this type of patient in depth, assessing specific executive capacities.

For group one, the results of the clinical effect seemed to be lower than for patients with long COVID, although the studies of higher methodological quality and with less risk of bias belong to group one. Group two, consisting of one controlled study (with under-sampling) and two quasi-experimental studies with only one intervention group, showed larger clinical effects, although these results should be considered with caution as both the variable “long COVID” and the higher risk of bias may affect the size of the effect assessed.

In comparison with traditional rehabilitation, only two studies carried out an analysis of efficacy [31,33], both of which position it as equally effective, and even superior in some parameters such as SGRQ [31]; although more studies are needed on this population, telerehabilitation already has a more than proven efficacy in other pathologies [18,43,44] as well as a much lower cost [45,46] and a good reception by patients [24,47]. These aspects make telerehabilitation a new trend in health sciences and, more specifically, boosted by the COVID-19 pandemic [48].

Another important aspect in telerehabilitation is adherence; only two studies reported data which are favorable. It seems that in the digital era in which we find ourselves, the implementation of this type of technology does not pose major problems; in other studies, with COVID-19 patients in the acute phase and with similar telerehabilitation methods, good results were also obtained in terms of adherence to telerehabilitation programs [49,50].

The main limitation of this review is the risk of bias; three of the five articles included did not include randomization or a control group, which greatly limits the level of evidence generated, and as a future perspective, studies with a higher quality methodological design should be conducted, especially on patients with “long COVID”.

## 5. Conclusions

Telerehabilitation through therapeutic exercise based on mixed protocols of aerobic, respiratory, and low-load strength exercises appears to be an effective and safe strategy for the recovery of short- and long-term post-COVID-19 sequelae. However, more randomized controlled studies are needed to ensure an adequate level of evidence.

## Figures and Tables

**Table 1:** Search Strategy.

**Web of Science**	(“long COVID” OR “post COVID”) AND exercise AND telerehabilitation (“long COVID” OR “post COVID”) AND physiotherapy AND telerehabilitation
**PubMed**	(“long COVID” OR “post COVID”) AND physiotherapy AND telerehabilitation (“long COVID” OR “post COVID”) AND exercise AND telerehabilitation
**Scopus**	(“long COVID” OR “post COVID”) AND exercise AND telerehabilitation “long COVID” OR “post COVID”) AND physiotherapy AND telerehabilitation
**PEDro**	“Long COVID” “Post COVID”

**Table 2:** PICOS Question.

**Population**	Post COVID-19 patients with symptoms.
**Intervention**	Different methods of therapeutic exercise involving the use of telerehabilitation.
**Comparison**	Difference between initial assessment and final assessment.
**Outcome**	Any physical parameter, scale, or test. Validated for evaluation purposes.
**Study Design**	Randomized controlled studies or longitudinal studies.

**Table 3:** Individual Quality Assessment.

*PEDro Scale (RCT)*	1	2		3		4	5		6		7		8	9		10		11	Total
Li et al., 2021	X	X		X		X	-		-		X		X	X		X		X	8/10
Ismael Palali et al., 2022	X	X		X		X	X		-		-		X	X		X		X	8/10
*JADAD Scale (RCT)*	1			2			3				4			5					Total
Li et al., 2021	X			X			-				X			X					4/5
Ismael Palali et al., 2022	X			X			-				-			X					3/5
*Newcastle–Ottawa Scale (Non-RCT)*	1		2		3			4		1		1			2		3		Total
Calvo-Paniagua et al., 2022	X		-		X			X		-		-			X		X		5/9
Colas et al., 2022	X		X		X			X		XX		-			X		X		8/9
Estebanez-Perez et al., 2022	X		-		X			X		-		-			X		X		5/9

## Data Availability

Not applicable.

## Appendix A

**Table A1:** Characteristics of Studies.

**Authors**	**Type of Study**	**Patients**	**Telerehabilitation Method**	**Intervention**	**Measures**	**Outcomes**
Jian’an Li et al. (2021) [32] Group one	Randomized controlled trial (*n* = 120)	Postdischarged patients with British Medical Research Council (mMRC) Dyspnoea Score of 2–3.	Unsupervised exercise program through smartphone app (RehabApp), teleconsultations 1 per week.	Control group (Short educational instructions) Intervention group (breathing control, thoracic expansion, AE, LMS); three to four sessions/week for 6 weeks.	6MWD (6MWT) Static Squad Test (LMS) SF-12 Spirometry: FEV1 (L) FVC (L) FEV1/FVC, MVV (L/min) (PEF) (L/s)	Intervention group show higher values in 6MWD, LMS and SF-12. No difference in pulmonary function.
Jose Calvo-Paniagua et al. (2022) [34] Group two	Quasi-experimental study (*n* = 68)	Fatigue and dyspnea as main post-COVID symptoms from at least three months after the infection.	Supervised video conference (Zoom)	A total of 3 sessions/week until 18 sessions (40 min/session) for 7 weeks. Program: • Sessions of sanitary education and posture ergonomics; • Respiratory control, diaphragmatic respiration, secretion clearance, respiratory muscles exercise; • AE, active mobilisations and motor control exercise.	MBDS, mMRC, SGRQ, 6MWD (6MWT), self-perceived exertion VAS, Sp02, HR	Significant improvements with large effect sizes in all outcomes at self perceived exertion, mMRC, SGRQ, increased Sp02 Increased walked distance (6MWD).
Claire Colas et al. (2022) [33] Group two	Nonrandomized controlled study (*n* = 17)	Post-COVID-19 fatigue for > 3 months of COVID-19 infection.	Supervised video conference (Cisco Webex Meetings)	Control group (traditional rehabilitation) and telerehabilitation group Three sessions/week for 4 weeks Program: • 45 min AE; • 15 min RE.	CFS-11, MBDS, VO2 max, MAP, VT1, FEV-1, (VE/VCO2) (PetCO2 rest) (PetCO2 recovery) 6MWD (6MWT) Handgrip test (Kg)	Reduction in fatigue, no difference between groups. No time*group interaction except PetCO2 at rest for control group. Significant time effect in aerobic parameters. Improvement in VO2 max in both groups. Handgrip not increased for any group.
Estebanez-Pérez et al. (2022) [35] Group two	Quasi-experimental study (*n* = 32)	Permanent symptoms three months after diagnosis of COVID-19 and that have persisted for at least 2 months	Supervised and personalised program through digital physiotherapy app (Physiotec) and supervised video conference	Three to five session/week for 4 weeks: • Sessions limited to one per day and 45–50 min at maximum, starting at 20–30 min. • Strength training, aerobic training with gradual increase of the intensity (5–10% per week). • Secretion and ventilatory techniques.	60secSTS, SPPB, Adherence (Physiotec)	Significant changes in the functional capacity of the 60secSTS and in SPPB. Good adherence (18 sessions out of 20).
Pehlivan et al. (2022) [31] Group one	Randomized controlled trial (*n* = 40)	Deterioration in physical functions in the first 4 weeks after medical discharge post COVID-19.	Supervised video conference (smartphone)	Three sessions/week for 6 weeks Telegroup: sessions supervised by physiotherapist; patient education, paced running/self-walking on the corridor, breathing exercises, active cycle of breathing technique, range of motion exercise, and standing squat. Control group: one session of exercise training and a brochure including similar exercises.	mMRC, pain and fatigue (VAS), TUG, SPPB, SGRQ, BDI,	mMRC, TUG, SGRQ, significantly improved in tele group. Control group only improved in VAS. SPPB no changues in any groups. Only SGRQ obtain significant differences between groups.
